# Efficacy and safety of intraseptal and periodontal ligament anaesthesia for extraction of upper lateral incisors and lower first premolars in patients with type 2 diabetes mellitus

**DOI:** 10.3389/froh.2026.1908426

**Published:** 2026-08-24

**Authors:** Vladimir Biocanin, Bozidar Brkovic, Momir Stevanovic, Milica Vasiljevic, Marija Biocanin, Dragica Stojic

**Affiliations:** 1 Department of Oral Surgery, Faculty of Dentistry in Pancevo, University of Business Academy in Novi Sad, Novi Sad, Serbia; 2 Department of Oral Surgery, School of Dental Medicine, University of Belgrade, Belgrade, Serbia; 3 Department of Dentistry, Faculty of Medical Sciences, University of Kragujevac, Kragujevac, Serbia; 4 Private Practice, Belgrade, Serbia; 5 Department of Pharmacology, School of Dental Medicine, University of Belgrade, Belgrade, Serbia

**Keywords:** articaine with epinephrine, computer-controlled local anesthetic delivery system (CCLADS), intraseptal anaesthesia (ISA), local anaesthesia, periodontal ligament anaesthesia (PLA), type 2 diabetes mellitus

## Abstract

**Introduction:**

The use of local anesthetics with vasoconstrictors in patients with Type 2 diabetes mellitus (DM) requires particular caution because of possible cardiovascular effects and increased susceptibility of diabetic nerve fibers to ischemic injury. Intraseptal anaesthesia (ISA) and periodontal ligament anaesthesia (PLA) may represent effective alternatives to conventional local anaesthesia in diabetic patients. This study aimed to compare the clinical efficacy and safety profiles of ISA and PLA administered with a computer-controlled local anesthetic delivery system (CCLADS) for extraction of maxillary lateral incisors and mandibular first premolars in patients with controlled Type 2 DM.

**Materials and methods:**

A total of 120 patients with controlled Type 2 DM were randomly allocated into either the ISA (*n* = 60) or PLA (*n* = 60) group using a computer-generated randomization sequence. All patients received 0.6 mL of 4% articaine with 1:100,000 epinephrine (Ar + Ep) delivered using CCLADS. Each group comprised 30 patients undergoing extraction of a maxillary lateral incisor and 30 patients undergoing extraction of a mandibular first premolar. Evaluated parameters included anesthetic success rate, onset time, duration of soft tissue anaesthesia, width of anesthetic field, systolic and diastolic blood pressure, mean arterial pressure, and heart rate before, during, and after anaesthesia administration.

**Results:**

Immediate onset of anaesthesia was achieved in all successful cases. ISA demonstrated a higher success rate than PLA, particularly for extraction of mandibular first premolars, although the difference was not statistically significant. ISA provided significantly longer duration of soft tissue anaesthesia and significantly wider distribution of the anaesthetic field than PLA for both investigated teeth. Cardiovascular parameters remained generally stable throughout the observation period in both groups. anaesthesia in blood pressure and heart rate were observed during and after anesthetic administration, without clinically relevant hemodynamic alterations.

**Conclusion:**

Both ISA and PLA administered with CCLADS were safe and clinically effective anesthetic techniques in patients with controlled Type 2 DM, without clinically relevant cardiovascular alterations. The longer duration and wider distribution of soft tissue anaesthesia achieved with ISA may support its use as a reliable option for tooth extraction in diabetic patients.

## 1 Introduction

Diabetes mellitus (DM) is a metabolic disorder characterized by an elevation in plasma glucose level and impaired secretion or action of insulin. Bearing in mind that DM primarily affects microcirculation leading to microangiopathy and peripheral neuropathy (1), patients with DM may be of special interest for the use of local anaesthetics with a vasoconstrictor. Impaired nerve fibers in DM appear to be more susceptible to ischemic injury induced by local anaesthetic solution (2). It has already been shown that nerve injury occurred when high doses of local anaesthetic were applied in diabetic rats (3). In addition, it has been proposed that adrenaline as part of local anaesthetic solution should not be used in large amounts in DM patients, because of its tendency to raise the blood glucose level (4–6).

Standard anaesthetic techniques in dentistry, such as infiltration or mandibular block anaesthesia, sometimes fail to produce effective pulp or soft tissue anaesthesia for different reasons, such as incorrect anaesthetic technique, anatomical variations, inflammation at the site of injection (7). It is well established that in such situations additional anaesthetic techniques, such as intraseptal (ISA) or intraperiodontal (PLA) anaesthesia, provide successful anaesthesia (8, 9). Both ISA and PLA could successfully be used as primary anaesthetic techniques in dentistry for periodontal surgery, placing retraction cords, simple extractions, and endodontic procedures (10–16). Having in mind that block anaesthesia in experimentally induced DM in rats could produce damage to nerve fibers (2), ISA and PLA could be suggested as alternative techniques.

Both intraseptal and periodontal ligament anaesthetic techniques are intraosseous, and their success primarily depends on the penetration of local anaesthetic through the alveolar bone. Among local anaesthetics, articaine is an anaesthetic with such good characteristics because it contains thiophene ring, which allows greater liposolubility (17–19), and consequently, better penetration through bone and other tissues (20, 21). Clinically, it is manifested by a higher anaesthetic success rate in anaesthetising mandibular and maxillary posterior teeth by buccal infiltration compared to lidocaine (22–24).

While vasoconstrictors administered with local anaesthetics may have minimal effects on healthy individuals, they may cause significant changes in cardiovascular parameters in cardiovascular patients (25). The stability of cardiovascular parameters regarding the use of local anaesthetics with epinephrine is of special relevance in DM patients, who usually suffer from cardiovascular diseases (26). For this reason, clinicians should be very careful to prevent intravascular application of local anaesthetics with epinephrine in DM patients. Having in mind that both ISA and PLA are intraosseous anaesthetic techniques, where an anaesthetic with a vasoconstrictor enters systemic circulation rapidly, elevation of blood pressure and heart rate is expected.

Today, the modern approach to local anaesthesia application to oral tissues is known as computer-controlled local anaesthetic delivery system (CCLADS). Controlled delivery approach enables precise localization of the drug adjacent to the place of action, which lowers the total dosage of anaesthetic drug and vasoconstrictor, a factor that may be of special relevance in DM patients. Data obtained from many trials showed that application of anaesthesia with CCLADS produced no changes in heart rate, compared to the high-pressure local anaesthetic injection (15, 16, 27). This could be the reason to use CCLADS rather than a high-pressure syringe in DM patients who usually suffer from cardiovascular diseases and where stability of cardiovascular parameters is of special relevance.

Despite the potential advantages of ISA and PLA in patients with DM, no previous clinical study has compared their efficacy and cardiovascular safety in patients with controlled Type 2 DM. This represents an important gap in the literature because these patients may be particularly susceptible to ischemic nerve injury and cardiovascular effects associated with local anaesthetic solutions containing vasoconstrictors. Therefore, the aim of this study was to compare the clinical efficacy and cardiovascular safety of ISA and PLA administered with a computer-controlled local anaesthetic delivery system for extraction of maxillary lateral incisors and mandibular first premolars in patients with controlled Type 2 DM.

## 2 Materials and methods

### 2.1 Study population

One hundred and twenty patients with controlled type 2 DM were enrolled in this single-centre, single-blind, randomized clinical study. Controlled type 2 DM was defined based on patients medical history, regular antidiabetic therapy, and confirmation of stable disease control from available medical documentation. The sample size was planned before the initiation of the study based on previous clinical data regarding the efficacy of intraseptal and periodontal ligament anaesthesia using a computer-controlled local anaesthetic delivery system. A total of 30 patients per subgroup was planned to enable statistical comparison between the two anaesthetic techniques for each investigated tooth type. Eligible participants were randomly assigned in a 1:1 ratio to receive either intraseptal anaesthesia or periodontal ligament anaesthesia. Randomization was performed using a computer-generated random allocation sequence prepared before patient recruitment. To ensure balanced allocation according to the tooth scheduled for extraction, randomization was stratified by tooth type (maxillary lateral incisor or mandibular first premolar). Allocation was implemented using sequentially numbered, opaque, sealed envelopes that were opened immediately before administration of the anaesthetic technique. All subjects signed an informed consent, after they were duly informed of the study protocol and the aims of the study. The study was approved by the Ethical Committee of the Faculty of Dentistry, University of Belgrade (No.36/3).

Subjects had to be between 35 and 77 years of age and have no contraindications to the administration of local anaesthetic and its associated vasoconstrictor. This age range was selected because type 2 DM predominantly affects middle-aged and older adults, allowing inclusion of a representative diabetic population while minimizing age-related variability associated with younger individuals. It also corresponded to the population with established type 2 DM available for recruitment during the study period. The inclusion criteria were: patients aged 35–77 years with controlled type 2 DM, an indication for extraction of a maxillary lateral incisor or mandibular first premolar, and no contraindications to the administration of articaine with epinephrine.

The exclusion criteria were: smoking, known or suspected alcohol or drug dependence, dental treatment within the previous 48 h, acute periodontal disease, acute dentoalveolar abscess, history of tooth trauma, and tooth sensitivity. Additionally, patients with uncontrolled cardiovascular diseases or other systemic conditions that could influence cardiovascular parameters or interfere with the assessment of anaesthetic efficacy were excluded from the study. All the patients underwent an oral hygiene protocol using 15 mL of 0.12% chlorhexidine mouth rinse for 30 s, 5 min before the injection, in order to reduce bacteria at the local anaesthetic injection sites. The tested teeth were mandibular first premolars and maxillary lateral incisors, planned for routine extraction.

### 2.2 Local anaesthesia

Diabetic patients were randomly divided into the two main groups: the first group received 0.6 mL 4% articaine with 1:100.000 epinephrine (Ar + Ep) (Septanest®, Septodont, France) for ISA (60 patients) and the second group received 0.6 mL of the same anaesthetic solution for PLA (60 patients). The dose of 0.6 mL Ar + Ep was selected based on the results of our previous dose-finding study in healthy individuals, which demonstrated that this volume was clinically effective and safe. Each group was subdivided into two groups of 30 patients for the extraction of upper lateral incisor and lower first premolar. There was no premedication or additional medication given during the period of investigation. Anaesthetic solution was injected with computer-controlled local anaesthetic delivery system (CCLADS) (Anaeject®, Septodont, France) with constant pressure, speed, and dose of approximately 0.005 mL per second. The application period was 60 s for a 0.3 mL dose on the mesial side of the tooth, with the same applied to the distal side. Two minutes before the injection of the ISA and PLA, a topical anaesthetic was applied at that area.

The site of needle insertion for the ISA was 2–3 mm below the tip of the interdental mesial and distal papilla, with 90° angulation of the needle to the papilla's surface, until contact with the bone. Blanching of the gingiva overlying bone with no leakage of the anaesthetic were indicators that the anaesthetic solution had been properly deposited. The site of needle insertion for the PLA was the region of gingival sulcus at 30° to the tooth's long axis at the bucomesial and bucodistal aspect of the root. A 30G short needle (Septodont®, Dental Needle, France), for both the ISA and PLA, was used. The anaesthetic techniques were performed by one of the participating dentists according to the allocated treatment. Owing to the nature of the interventions, the operator could not be blinded to the anaesthetic technique. However, assessment of anaesthetic efficacy and all outcome measures was performed by an independent investigator who was not involved in treatment allocation and was blinded to group assignment.

### 2.3 Parameters of local anaesthesia

The anaesthetic efficacy (success rate), onset time, width of the anaesthetic field, and duration of soft tissue anaesthesia were assessed by the same independent investigator, who was blinded to treatment allocation. The tooth was extracted 5 min after application of the anaesthesia. Anaesthesia was considered successful when the patient did not feel any pain during extraction, and marked zero on the VAS scale. If the patient felt pain during extraction, the extraction process was immediately stopped, standard infiltration or mandibular block anaesthesia (1.8 mL of Ar + Ep) were applied and extraction was completed.

Onset time for anaesthesia was defined as the time, in seconds, from completion of the injection to the time at which profound anaesthesia of the adjacent gingiva was established. We assessed duration of soft tissue anaesthesia at 5-min intervals. Duration of soft tissue anaesthesia was recorded by negative response to pinprick testing until the moment when the patient felt sharp pain during pinprick testing, in the region of buccal attached gingiva. We used 27-gauge sterile needle (MonoJect®, Dental Needle, Mansfield, USA) for pinprick testing. Pinprick testing was performed directly until contact with the periosteum occurred. The width of the anaesthetic field, expressed in millimetres, was measured in the region of buccal attached gingiva, lingual attached gingiva, palatal and oral mucosa, 10 min after successful anaesthesia was obtained, by flexible ruler and pinprick testing.

### 2.4 Evaluation of cardiovascular parameters

Systolic blood pressure (SP), diastolic blood pressure (DP), mean arterial pressure (MP), and heart rate (HR) were recorded by electrocardiogram monitor (Datex-Engstrom AS/3, Helsinki, Finland) at 6 times: 5 min prior to anaesthesia, during anaesthetic injection and 5, 10, 15 and 30 min after administering anaesthesia.

### 2.5 Statistical analysis

Statistical analysis was performed using statistical software SPSS, version 27.0. Variables with normal distribution were presented as mean ± SD, whereas variables that were not normally distributed were presented as median (interquartile range). The Chi-Square test was used to determine differences in gender and success rate between groups. Age and weight of patients, duration of soft tissue anaesthesia and width of anaesthetic field in the region of attached gingiva and oral mucosa of upper lateral incisor and lower first premolar, and lingual attached gingiva of lower first premolar were analyzed using independent-samples *t*-test. Mann–Whitney test was used to determine differences in width of anaesthetic field of palatal mucosa. Comparisons were considered significant at *p* < 0.05.

## 3 Results

**Figure 1:**
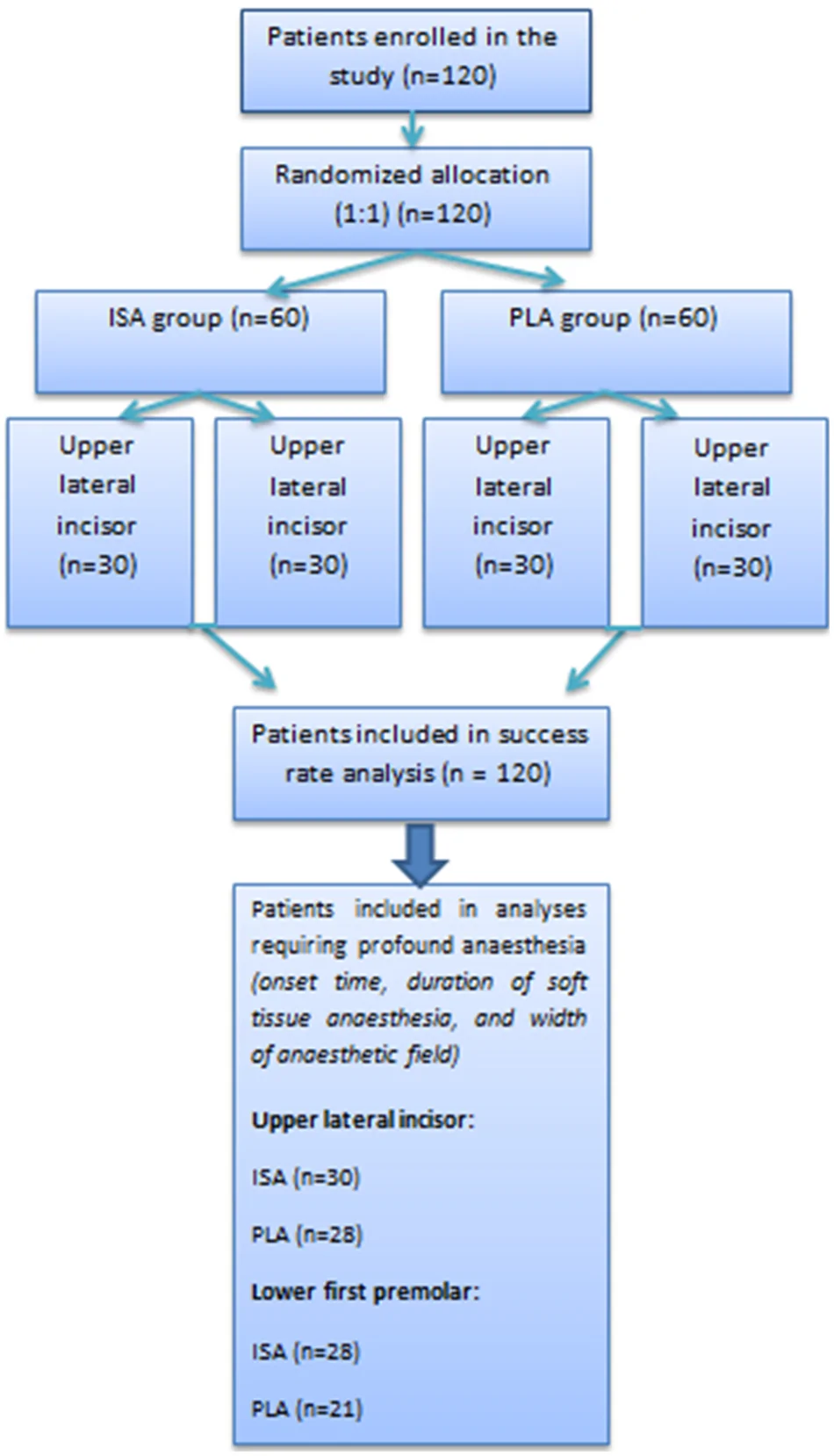
Flow diagram of patient allocation and analysis in the study.

The flow of patients through the study and the number of patients included in each analysis are presented in Figure 1.

The demographic characteristics of the patients are presented in Table 1. No statistically significant differences were observed between the groups regarding age, sex, or weight. The study included 56 (46.67%) male and 64 (53.33%) female patients with type 2 DM.

**Table 1:** Demographic characteristics of patients with type 2 DM undergoing extraction of the upper lateral incisor and lower first premolar using ISA and PLA anaesthesia.

	Upper lateral incisor		Lower first premolar	
	ISA	PLA	ISA	PLA
	Doses (articaine + epinephrine)			
	0.6 mL	0.6 mL	0.6 mL	0.6 mL
*N*	30	30	30	30
Age^a^ (*X* ± SD)	58.1 ± 12.5	60.0 ± 7.3	59.7 ± 11.4	61.5 ± 6.9
Male/female^b^	13/17	15/15	14/16	14/16
Weight/kg^a^ (*X* ± SD)	81.0 ± 14.3	76.7 ± 10.7	79.9 ± 16.4	76.0 ± 10.0

*N*, number of anaesthetised teeth.

a No statistically significant differences were observed between groups (*t*-test, *p* > 0.05). Independent samples *t*-test: age (*p* = 0.47 and *p* = 0.46), weight (*p* = 0.19 and *p* = 0.28) for maxillary lateral incisor and mandibular first premolar, respectively.

b No statistically significant differences were observed between groups (Chi-square test, *p* > 0.05). Chi-square test: sex distribution (*p* = 0.60 and *p* = 1.00 for maxillary lateral incisor and mandibular first premolar, respectively).

### 3.1 Parameters of local anaesthesia

The success rates of ISA and PLA were evaluated in all 30 patients in each investigated group. Complete success of anaesthesia (100%) was obtained in the group of ISA for the extraction of upper lateral incisor (Table 2). Although success rate of ISA (93%) for the extraction of the lower first premolar was higher than with PLA (70%), the difference was not statistically significant (Table 2; *p* > 0.05).

**Table 2:** Success rate of bone and gingival anaesthesia in patients with type 2 DM after ISA and PLA anaesthesia, defined as painless extraction (VAS score = 0).

	ISA	PLA
	Doses (articaine + epinephrine)	
	0.6 mL	0.6 mL
Success rate	100.0%	93.3%
Upper lateral incisor (*N*)	(30/30)	(28/30)
Success rate	93.3%	70.0%
Lower first premolar (*N*)	(28/30)	(21/30)

Chi-square test.

Only data for the subjects who achieved profound anaesthesia (30 out of 30 and 28 out of 30 subjects in the ISA for the extraction of upper lateral incisor and lower first premolar, respectively; and 28 out of 30 and 21 out of 30 subjects in the PLA for the extraction of upper lateral incisor and lower first premolar, respectively) were available to calculate onset, duration, and width of the anaesthetic field. Soft tissue anaesthesia in all patients was achieved immediately after the end of application.

Duration of soft tissue anaesthesia was significantly longer with ISA than with PLA, both around upper lateral incisor and lower first premolar (Table 3; *p* < 0.05).

**Table 3:** Duration of soft tissue anaesthesia around the upper lateral incisor and lower first premolar in patients with type 2 DM after ISA and PLA.

Duration of soft tissue anaesthesia (min)	ISA	PLA
	Doses (articaine + epinephrine)	
	0.6 mL	0.6 mL
Upper lateral incisor		
(*X* ± SD)	60.5 ± 14.2^a^	46.2 ± 14.3
(*N*)	(30)	(28)
Lower first premolar		
(*X* ± SD)	55.8 ± 14.3^a^	45.8 ± 11.8
(*N*)	(28)	(21)

*N*, number of patients with successful anaesthesia included in the analysis.

a *p* < 0.05 (*t*-test). Independent samples *t*-test: *p* = 0.001 for upper lateral incisor and *p* = 0.011 for lower first premolar.

The width of the anaesthetic field in the region of the buccal attached gingiva, oral mucosa, palatal mucosa, and lingual attached gingiva was significantly higher in ISA groups compared to PLA (Tables 4, 5; *p* < 0.05).

**Table 4:** Width of anaesthetic field in attached gingiva and oral/palatal mucosa around the upper lateral incisor in patients with type 2 DM after ISA and PLA.

Width of anaesthetic field	ISA	PLA
	Doses (articaine + epinephrine)	
	0.6 mL	0.6 mL
Attached gingiva/mm		
(*X* ± SD)	34.4 ± 12.2^a^	19.9 ± 5.6
(*N*)	(30)	(28)
Oral mucosa/mm		
(*X* ± SD)	30.8 ± 11.2^a^	16.8 ± 5.0
(*N*)	(30)	(28)
Palatal mucosa/mm		
(*X* ± SD)	18.2 ± 8.6^b^	11.8 ± 4.0
(*N*)	(30)	(28)

*N*, number of patients with successful anaesthesia included in the analysis.

a *p* < 0.001 (*t*-test).

b *p* < 0.05 (Mann–Whitney *U*-test).

**Table 5:** Width of anaesthetic field in attached gingiva and oral/lingual mucosa around the lower first premolar in patients with type 2 DM after ISA and PLA.

Width of anaesthetic field	ISA	PLA
	Doses (articaine + epinephrine)	
	0.6 mL	0.6 mL
Attached gingiva/mm		
(*X* ± SD)	34.2 ± 8.5^a^	21.4 ± 6.3
(*N*)	(28)	(21)
Oral mucosa/mm		
(*X* ± SD)	33.2 ± 8.7^a^	18.5 ± 5.3
(*N*)	(28)	(21)
Lingval attached gingiva/mm		
(*X* ± SD)	24.1 ± 9.9^a^	16.2 ± 6.4
(*N*)	(28)	(21)

*N*, number of patients with successful anaesthesia included in the analysis.

a *p* < 0.05 (*t*-test). Independent samples *t*-test: attached gingiva (*p* < 0.001), oral mucosa (*p* < 0.001), and lingual attached gingiva (*p* = 0.002).

### 3.2 Parameters of cardiovascular function

anaesthesiaanaesthesiaanaesthesia Baseline cardiovascular parameters were comparable between the ISA and PLA groups, with no statistically significant differences in systolic blood pressure (129.3 ± 20.7 vs. 125.9 ± 17.9 mmHg, *p* = 0.406), diastolic blood pressure (72.7 ± 10.0 vs. 73.5 ± 8.6 mmHg, *p* = 0.664), mean arterial pressure (101.2 ± 14.6 vs. 100.2 ± 12.6 mmHg, *p* = 0.723), or heart rate (77.8 ± 13.4 vs. 81.7 ± 11.9 bpm, *p* = 0.158). During anaesthetic administration, small but statistically significant increases compared with baseline values were observed in the ISA group for systolic blood pressure (*p* < 0.001), diastolic blood pressure (*p* = 0.004), mean arterial pressure (*p* < 0.001), and heart rate (*p* = 0.039). In the PLA group, no statistically significant changes from baseline were observed in systolic blood pressure, diastolic blood pressure, or mean arterial pressure (all *p* > 0.05), whereas heart rate showed a small but statistically significant increase (*p* = 0.020). Despite statistical significance, the magnitude of these changes was small and was not considered clinically relevant.

#### 3.2.1 Maxillary lateral incisor

**Figure 2:**
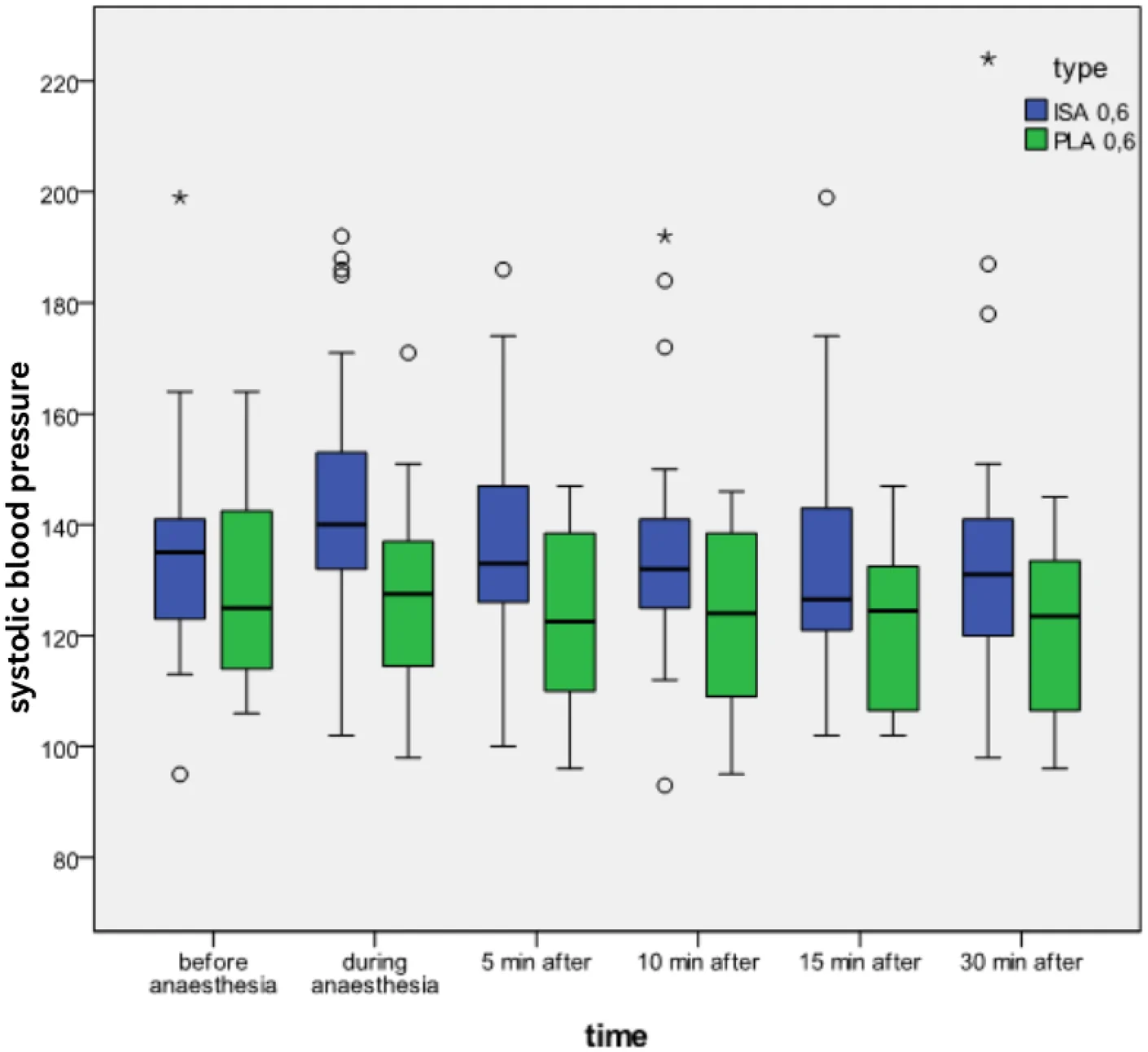
Changes in systolic blood pressure at different time intervals following ISA and PLA anaesthesia of the upper lateral incisor in patients with type 2 DM. * Blue box plots represent ISA (0.6 mL), whereas green box plots represent PLA (0.6 mL). Measurements were obtained before anaesthesia, during injection, and 5, 10, 15, and 30 min after anaesthesia administration.

Figure 2 demonstrates changes in systolic blood pressure during and after administration of two local anaesthesia techniques used for extraction of the maxillary lateral incisor. Systolic blood pressure values remained relatively stable throughout the observation period in both groups. Compared with baseline, a small but statistically significant increase in systolic blood pressure was observed during anaesthetic administration in the ISA group (*p* < 0.001), whereas no significant change was observed in the PLA group (*p* > 0.05). However, median values tended to be slightly higher in the ISA group compared with the PLA group at most measurement intervals. Significant differences between the groups were observed before anaesthesia administration and at 10 and 30 min post-administration. Moderate interindividual variability was observed, without major hemodynamic fluctuations during the procedure.

**Figure 3:**
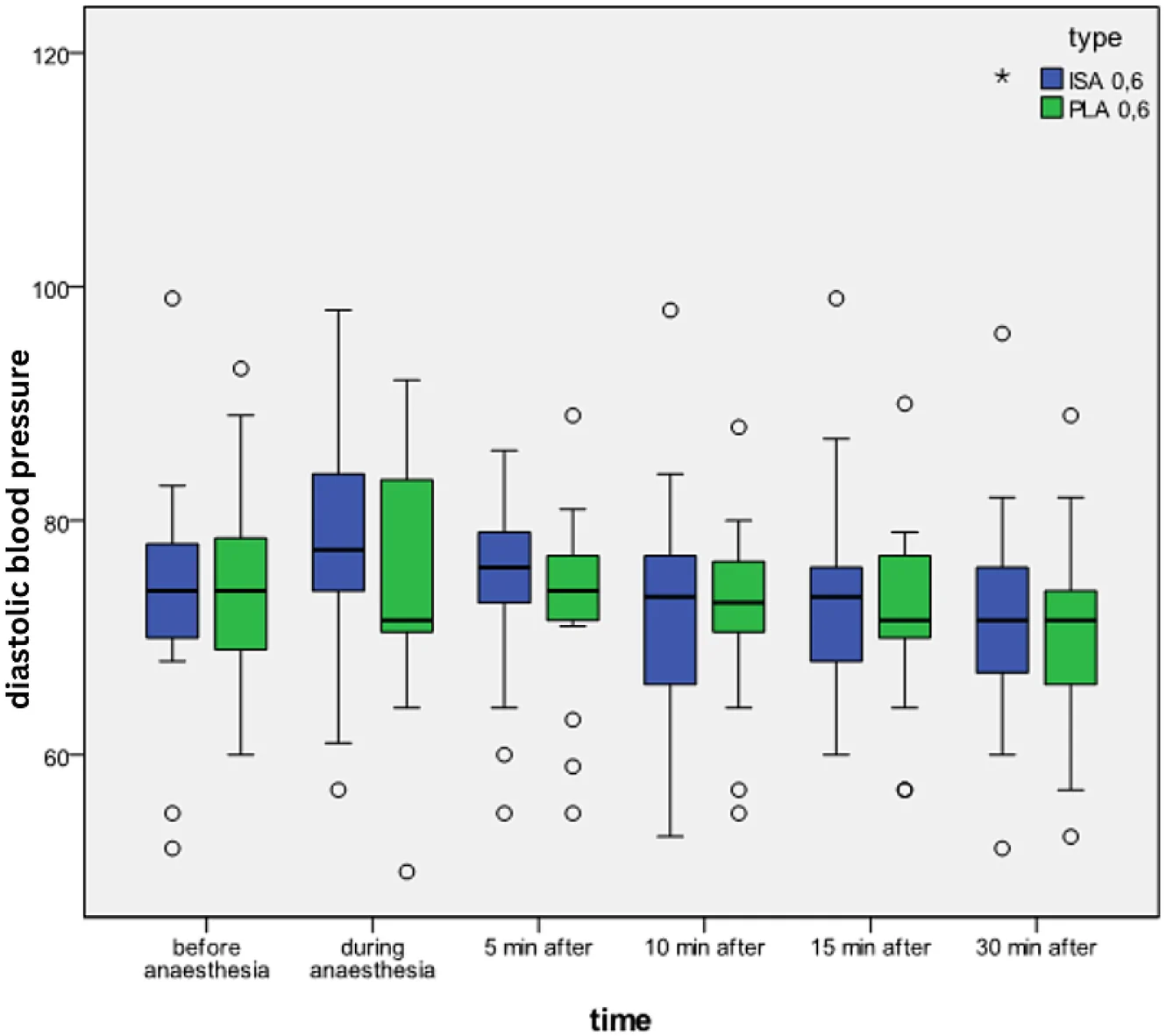
Changes in diastolic blood pressure at different time intervals following ISA and PLA anaesthesia of the upper lateral incisor in patients with type 2 DM. * Blue box plots represent ISA (0.6 mL), whereas green box plots represent PLA (0.6 mL). Measurements were obtained before anaesthesia, during injection, and 5, 10, 15, and 30 min after anaesthesia administration.

Changes in diastolic blood pressure during and after administration of the two local anaesthesia techniques used for extraction of the maxillary lateral incisor are shown in Figure 3. Diastolic blood pressure values remained relatively stable throughout the observation period in both groups. Compared with baseline, diastolic blood pressure increased slightly during anaesthetic administration in the ISA group (*p* = 0.004), whereas no statistically significant change was observed in the PLA group (*p* > 0.05). Median values were comparable between the groups at most measurement intervals, although slightly higher values were observed in the ISA group during anaesthesia administration and in the early post-anesthetic period. A statistically significant difference between the groups was observed 30 min after anaesthesia administration. Moderate interindividual variability was present, without clinically relevant hemodynamic changes during the procedure.

**Figure 4:**
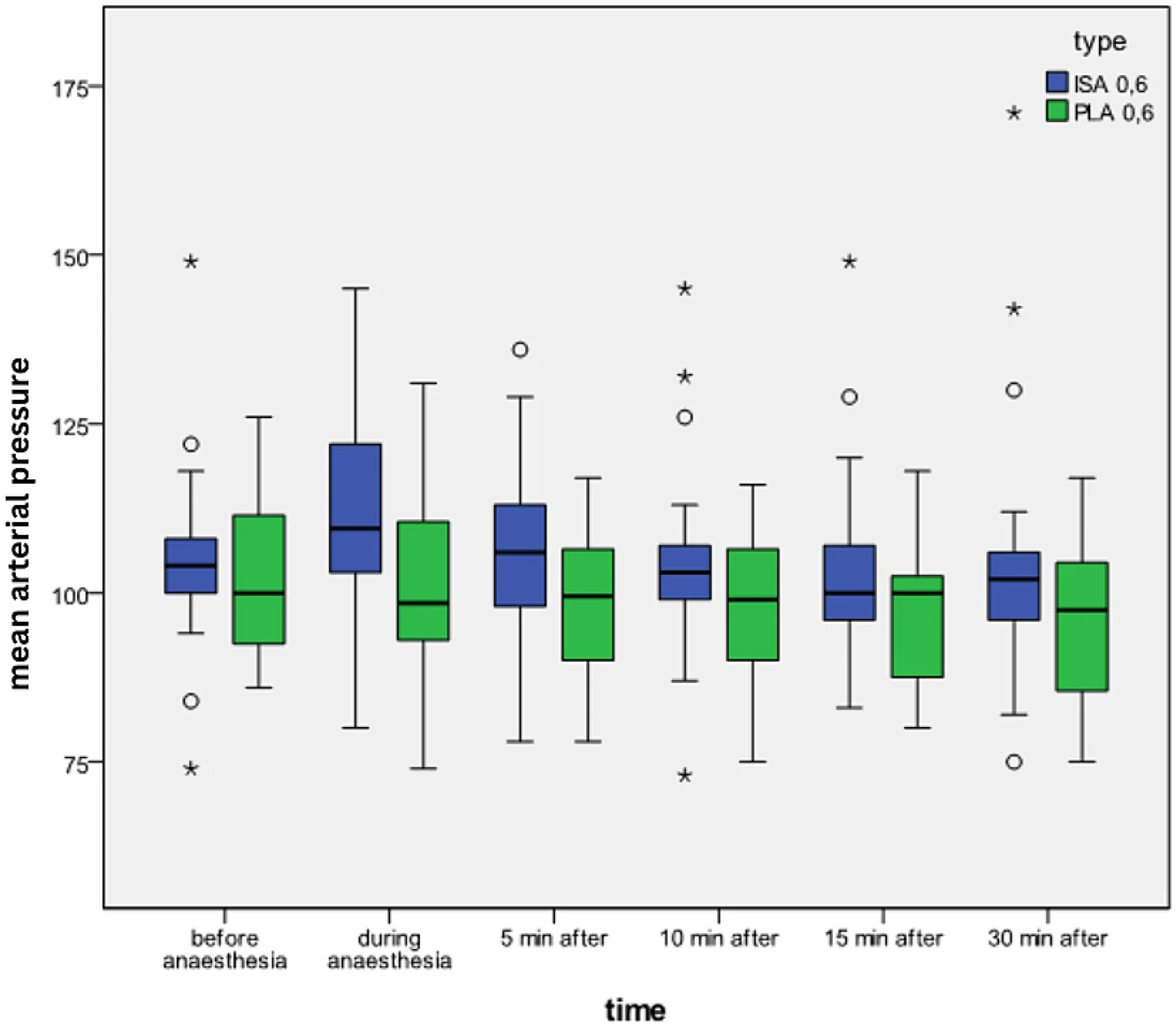
Changes in mean arterial pressure at different time intervals following ISA and PLA anaesthesia of the upper lateral incisor in patients with type 2 DM. * Blue box plots represent ISA (0.6 mL), whereas green box plots represent PLA (0.6 mL). Measurements were obtained before anaesthesia, during injection, and 5, 10, 15, and 30 min after anaesthesia administration.

Mean arterial pressure values remained relatively stable throughout the observation period in both groups during extraction of the maxillary lateral incisor (Figure 4). Compared with baseline, a small but statistically significant increase in mean arterial pressure was observed during anaesthetic administration in the ISA group (*p* < 0.001), whereas no statistically significant change was detected in the PLA group (*p* > 0.05). However, median values tended to be slightly higher in the ISA group compared with the PLA group. Statistically significant differences between the groups were observed before anaesthesia administration, as well as 10, 15, and 30 min after anaesthesia administration. Moderate interindividual variability was observed, without marked hemodynamic alterations during the procedure.

**Figure 5:**
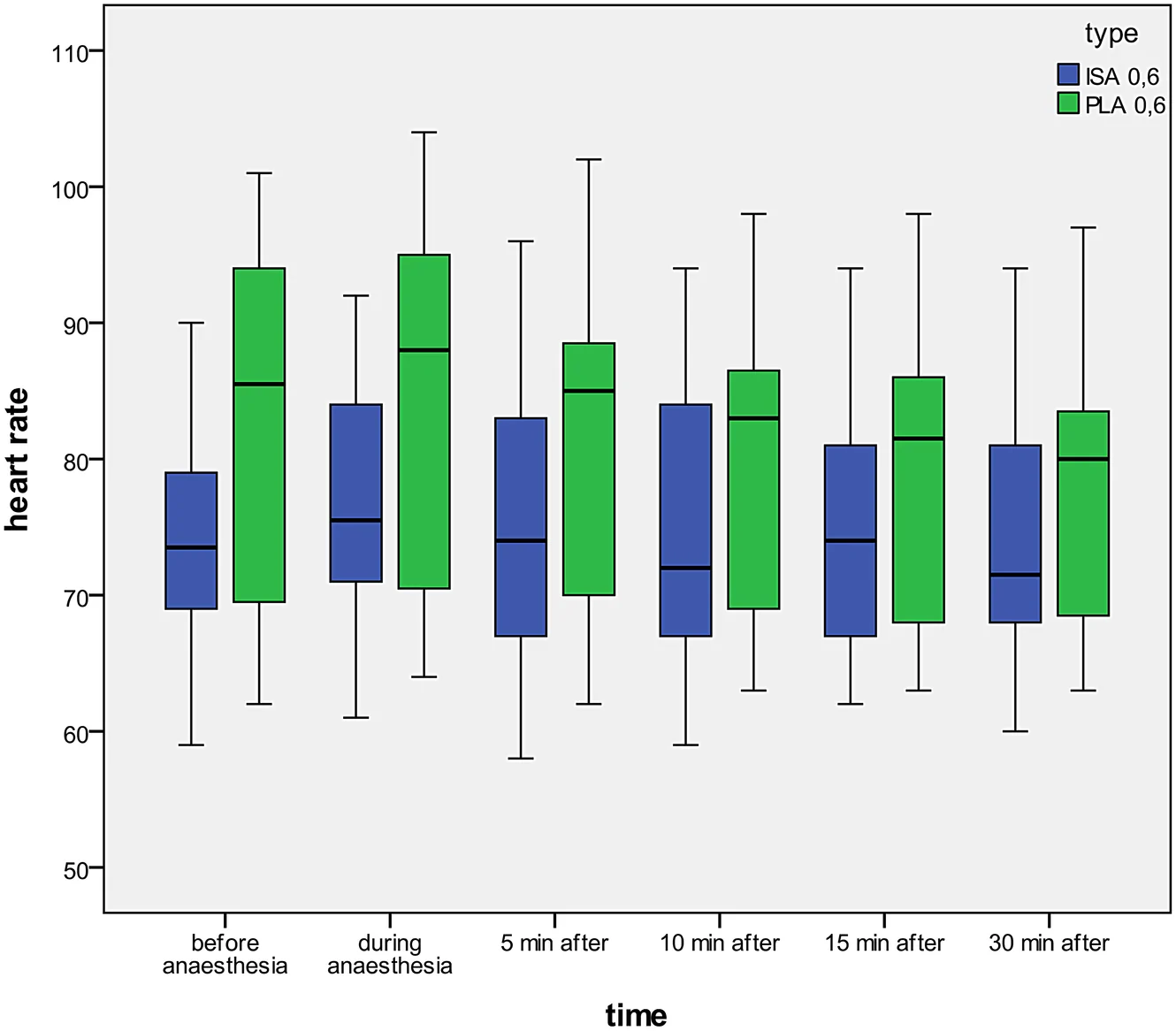
Changes in heart rate at different time intervals following ISA and PLA anesthesia of the upper lateral incisor in patients with type 2 DM. * Blue box plots represent ISA (0.6 mL), whereas green box plots represent PLA (0.6 mL). Measurements were obtained before anaesthesia, during injection, and 5, 10, 15, and 30 min after anaesthesia administration.

Heart rate measurements showed no major fluctuations throughout the observation period in either group during extraction of the maxillary lateral incisor (Figure 5). Compared with baseline, heart rate increased slightly but significantly during anaesthetic administration in both the ISA (*p* = 0.039) and PLA (*p* = 0.020) groups. However, these changes were transient and were not considered clinically relevant. However, slightly higher median values were consistently observed in the PLA group compared with the ISA group. In both groups, heart rate values showed a mild increase during anesthetic administration, followed by a gradual return toward baseline values during the observation period. Greater variability was observed in the PLA group, without marked cardiovascular alterations during the procedure.

#### 3.2.2 Mandibular first premolar

**Figure 6:**
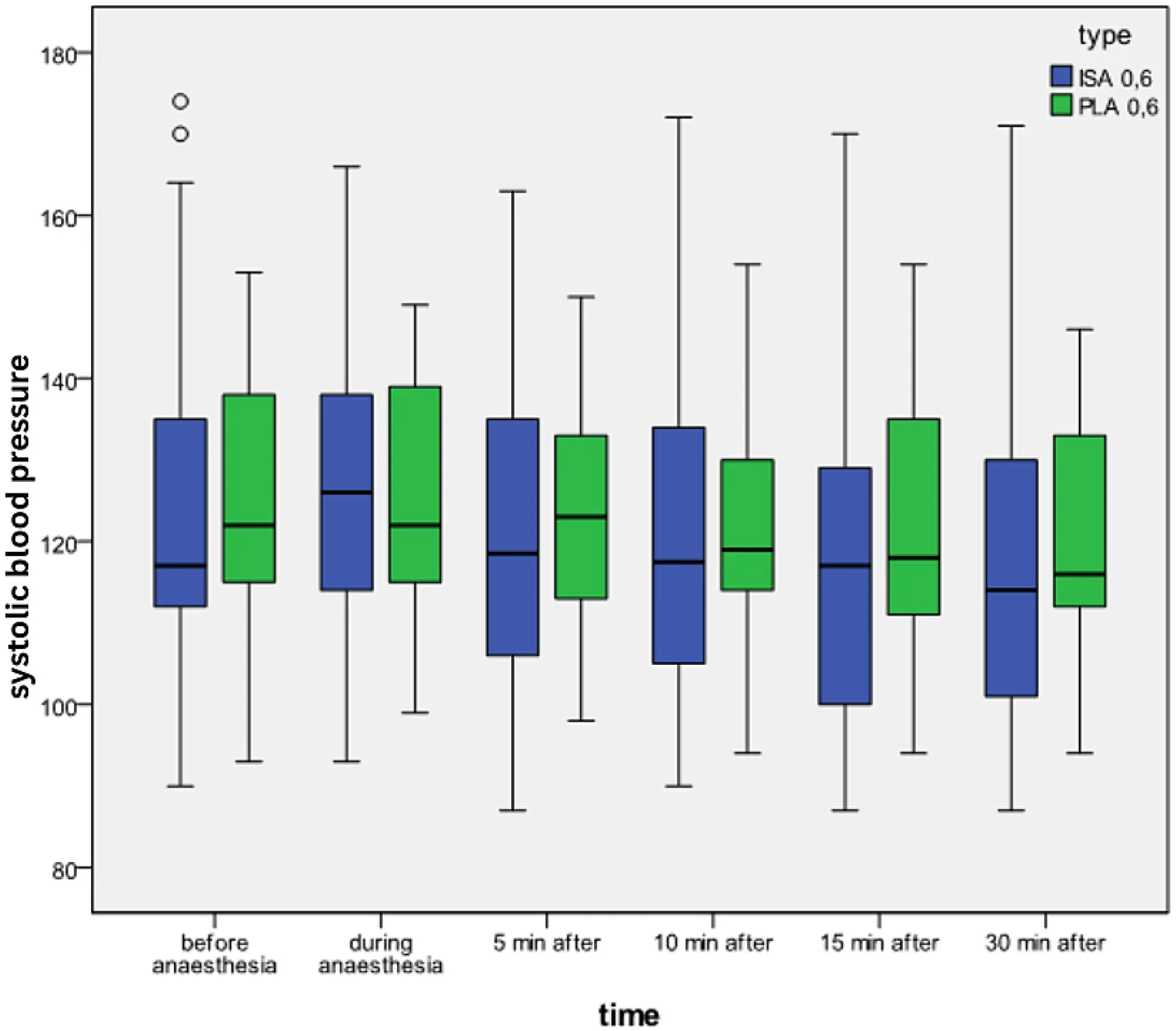
Changes in systolic blood pressure at different time intervals following ISA and PLA anaesthesia of the lower first premolar in patients with type 2 DM. * Blue box plots represent ISA (0.6 mL), whereas green box plots represent PLA (0.6 mL). Measurements were obtained before anaesthesia, during injection, and 5, 10, 15, and 30 min after anaesthesia administration.

No major changes in systolic blood pressure were observed throughout the observation period in both groups during extraction of the mandibular first premolar (Figure 6). Compared with baseline values, only minor transient variations in systolic blood pressure were observed during anaesthetic administration, with no clinically relevant increase in either anaesthesia group. However, slightly higher median values were generally observed in the PLA group compared with the ISA group. In both groups, systolic blood pressure showed a mild increase during anesthetic administration, followed by a gradual return toward baseline values during the observation period. Moderate interindividual variability was observed, particularly in the ISA group, without marked hemodynamic alterations during the procedure.

**Figure 7:**
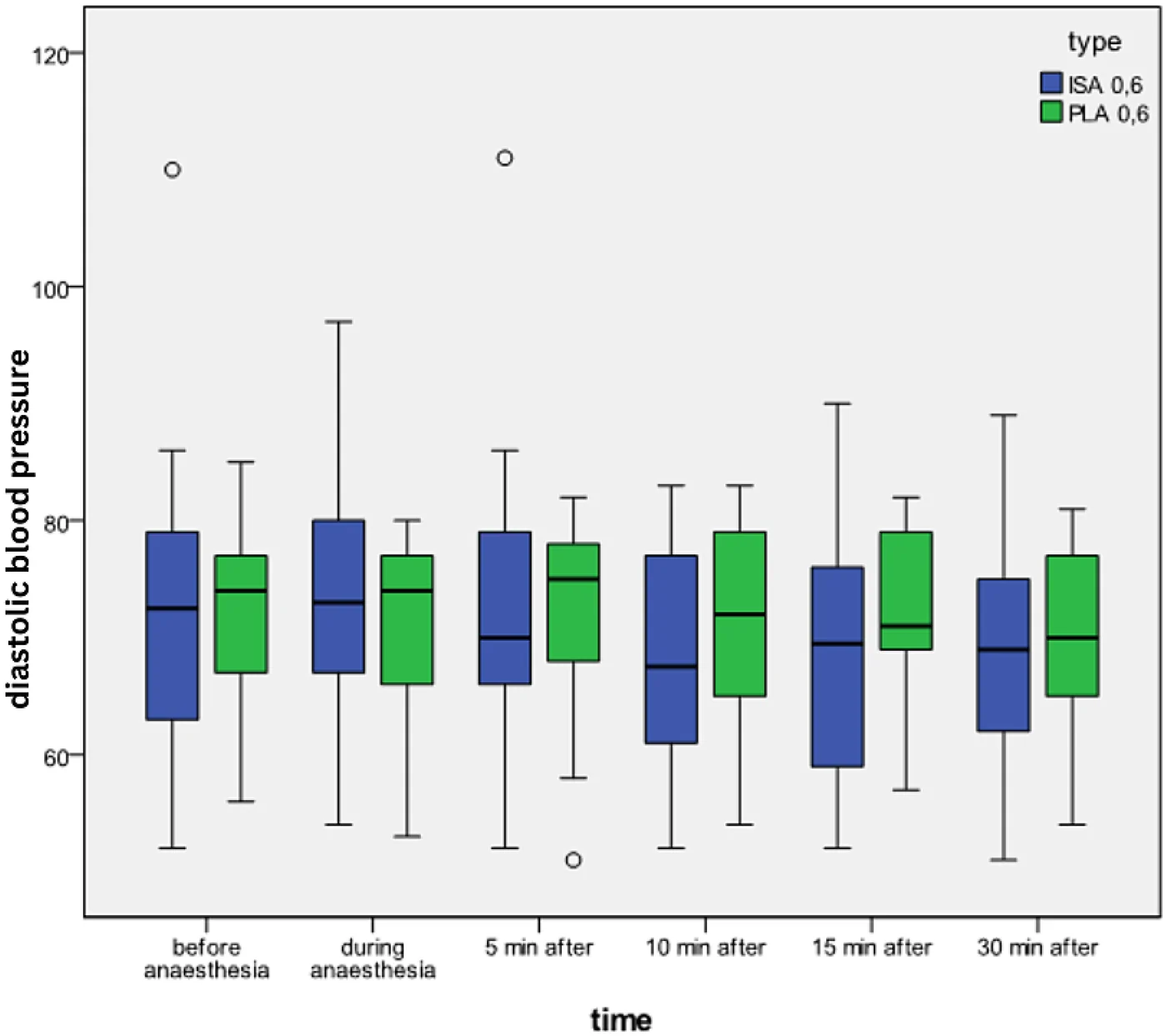
Changes in diastolic blood pressure at different time intervals following ISA and PLA anaesthesia of the lower first premolar in patients with type 2 DM. * Blue box plots represent ISA (0.6 mL), whereas green box plots represent PLA (0.6 mL). Measurements were obtained before anaesthesia, during injection, and 5, 10, 15, and 30 min after anaesthesia administration.

Diastolic blood pressure values remained relatively stable throughout all measured time intervals in both anaesthesia groups during extraction of the mandibular first premolar (Figure 7). Compared with baseline, only minor fluctuations were observed during anaesthetic administration, without clinically relevant changes in either group. A mild decrease in median values was observed 10 min after anaesthesia administration in the ISA group. However, no statistically significant differences between the groups were detected at any time point.

**Figure 8:**
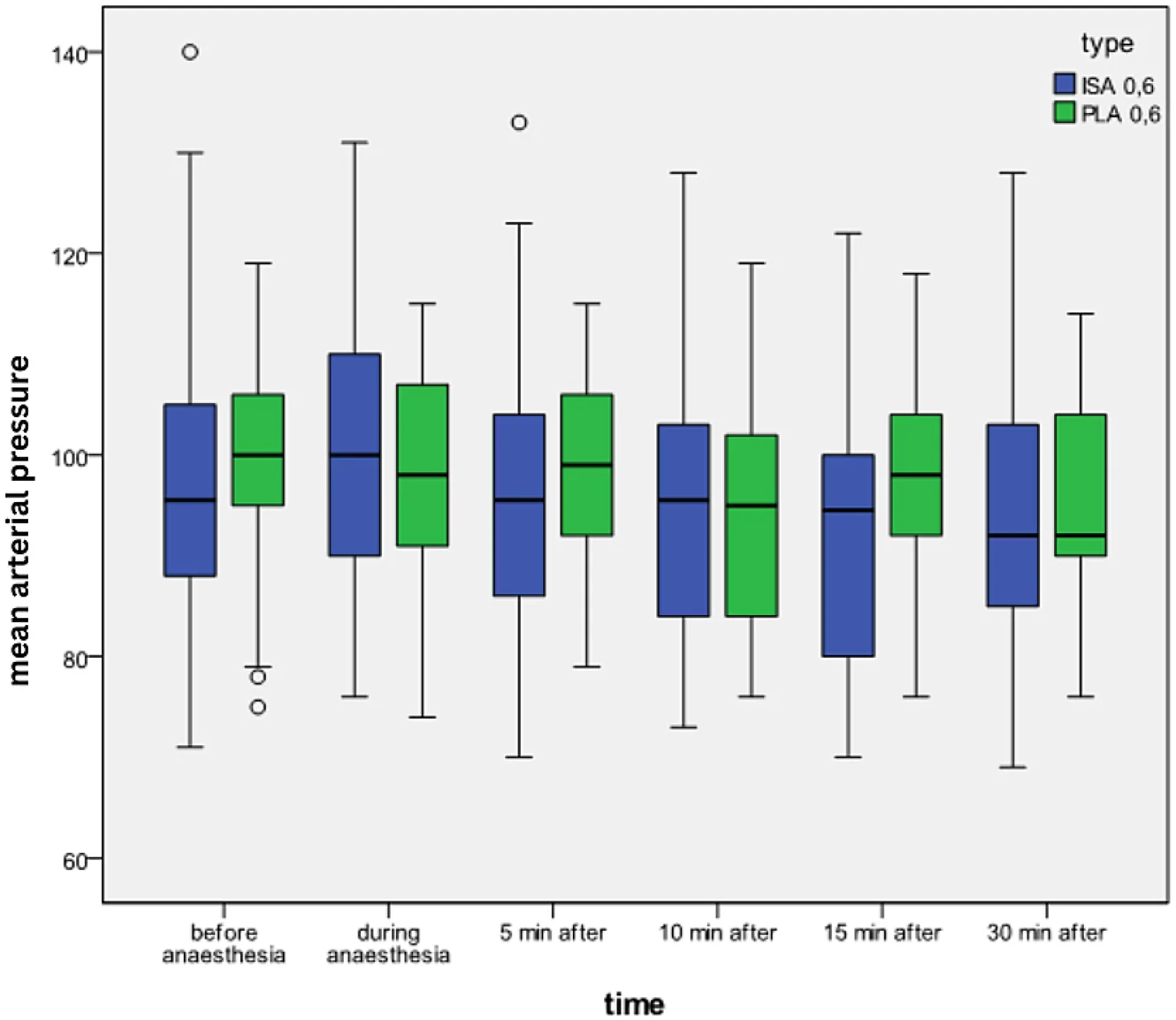
Changes in mean arterial pressure at different time intervals following ISA and PLA anaesthesia of the lower first premolar in patients with type 2 DM. * Blue box plots represent ISA (0.6 mL), whereas green box plots represent PLA (0.6 mL). Measurements were obtained before anaesthesia, during injection, and 5, 10, 15, and 30 min after anaesthesia administration.

Mean arterial pressure values remained relatively stable throughout all observed time intervals in both anaesthesia groups during extraction of the mandibular first premolar (Figure 8). Compared with baseline, only minor variations were observed during and after anaesthetic administration, with slightly higher median values during the early observation period, particularly in the PLA group. These changes were small and no statistically significant differences between the groups were observed at any measurement point.

**Figure 9:**
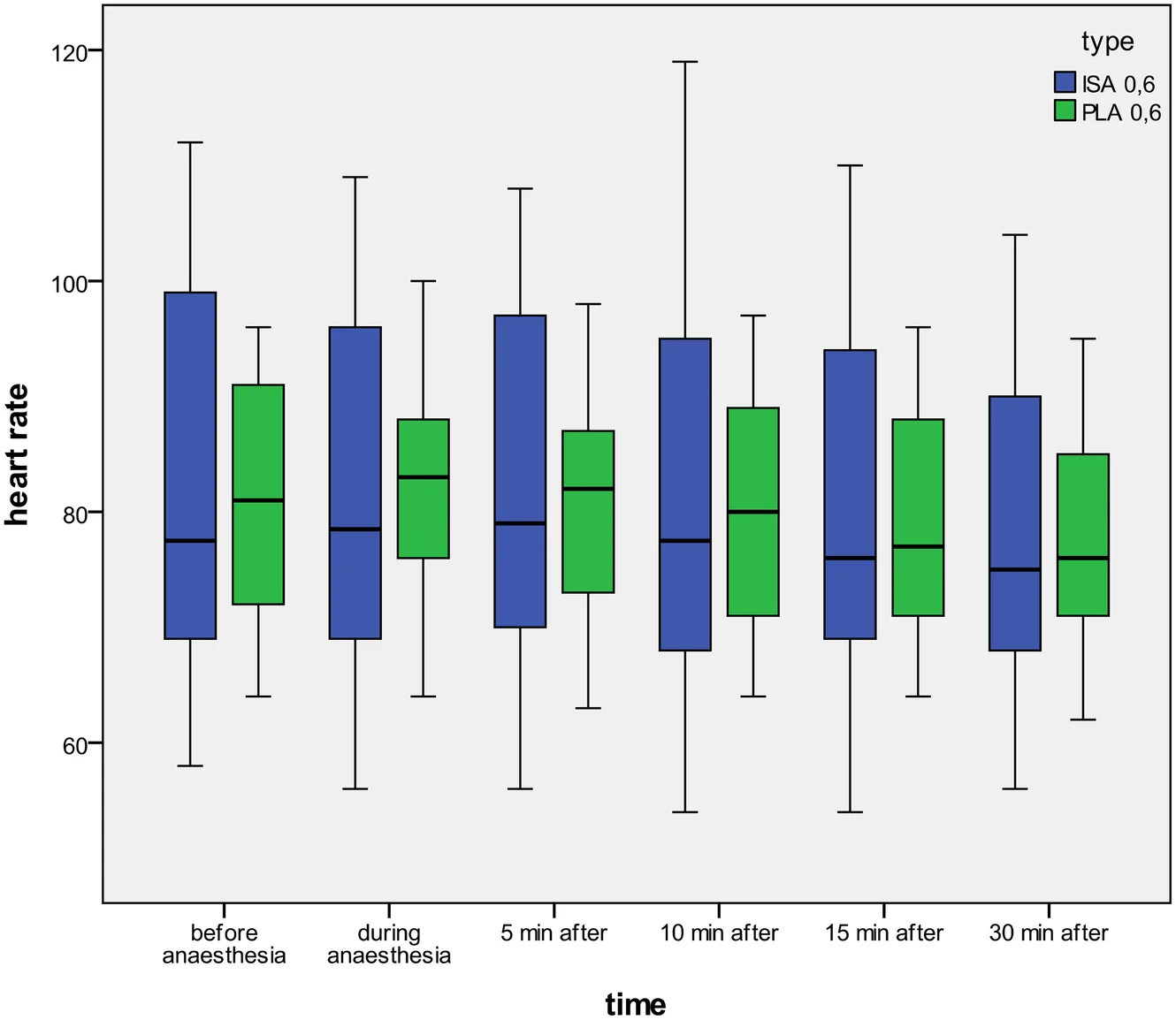
Changes in heart rate at different time intervals following ISA and PLA anesthesia of the lower first premolar in patients with type 2 DM. * Blue box plots represent ISA (0.6 mL), whereas green box plots represent PLA (0.6 mL). Measurements were obtained before anaesthesia, during injection, and 5, 10, 15, and 30 min after anaesthesia administration.

Heart rate values showed only minor variations over the observed time intervals in both anaesthesia groups during extraction of the mandibular first premolar (Figure 9). Compared with baseline, small transient changes in heart rate were observed during anaesthetic administration, without clinically relevant alterations. Median heart rate remained relatively consistent before, during, and after anaesthesia administration, with slightly higher values observed in the PLA group at several time points. Nevertheless, no statistically significant differences between the groups were identified during the observation period.

## 4 Discussion

The main advantage of ISA and PLA compared with standard anaesthetic techniques offers a lower dosage of local anaesthetic with vasoconstrictor required for successful anaesthesia (28). This may be of special benefit when a DM patient is a candidate for local anaesthesia.

The period of time from deposition of local anaesthetic to the moment when complete anaesthesia was achieved was characterised as onset time. The most important factors defining onset time are the concentration and pH of the local anaesthetic solution (29). This study showed that onset time for both ISA and PLA was immediate after the completion of the CCLADS application. The duration of anaesthetic application with CCLADS was 2 min. In addition, articaine, as a 4% anaesthetic solution, which we used in this study, enables faster induction of anaesthesia and consequently a shorter onset time compared with a 2% anaesthetic solution such as lidocaine.

Success rate of local anaesthesia depends on different factors, such as adequate anaesthetic technique, adequate amount of local anaesthesia, inflammation to the site of injection, intravascular injection and anatomical variations (30). There were no statistically significant differences in success rate between ISA and PLA for both upper lateral incisor and lower first premolar. This may be explained by the fact that both upper lateral incisor and lower first premolar have similar volume of periodontal ligament. Clinically acceptable success rate of anaesthesia was obtained with the ISA using 0.6 mL of Ar + Ep for both upper lateral incisor (100%) and lower first premolar (93.3%) and with PLA for upper lateral incisor (93.3%), in contrast to the PLA for lower first premolar (70%). These findings are in agreement with the study of Brkovic et al. (31), who reported a success rate of 91.4% for the extraction of upper lateral incisors with PLA, but using a higher dose, 0.8 mL of 2% Lidocaine+1:100.000 Ep, in healthy individuals. Similar success rate of PLA in our study in DM patients obtained with a smaller amount of anaesthetic solution (0.6 mL), compared with that in healthy individuals (31) for the extraction of upper lateral incisors, could be explained by firstly, reduced amount of anaesthetic solution needed for diabetic patients compared to healthy individuals (32), and secondly, characteristics of articaine—its higher liposolubility and better penetration through nerve membrane (33). On the other hand, higher success rate of ISA, obtained in our study in DM patients, compared to success of ISA in healthy individuals (31) could possibly be attributed to DM neuropathy with accompanying decreased sensation (34) and higher sensitivity of diabetic nerve fibers to local anaesthetics (35) as well as articaine’s better penetration through bone than lidocaine (36).

Duration of local anaesthesia primarily depends on diffusion capacity, amount of local anaesthetic at the site of injection and its binding to plasma proteins (17). The results of this study showed longer duration of anaesthesia and wider area of soft tissue anaesthesia with ISA compared to PLA, in DM patients. Shorter duration of anaesthesia in PLA compared to ISA groups could be explained by the fact that injection site for PLA is located in the first third of the periodontal ligament, where periodontal blood supply is mostly localized (37, 38), and, presumably, the rapid absorption of anaesthetic solution occurs. In addition, the presence of fenestrated capillaries in periodontium with greater permeability (39) may also be responsible for rapid absorption of anaesthetic solution with PLA, and consequently shorter duration of anaesthesia. This is especially pronounced in DM patients, where the absorption of anaesthetic solution occurs even faster, due to inflammation of periodontium. Furthermore, it has been shown that DM has a negative effect on the periodontal inflammation (40, 41). Likewise, dissociation of local anaesthetic in inflamed periodontal tissue disables the effectiveness of PLA. On the other hand, the injection site of the ISA involves the interdental septum, and, in that way, ISA bypasses inflamed periodontal tissue, allowing deposition of the entire amount of anaesthetic solution directly into the alveolar bone. This could explain the significantly longer duration of anaesthesia with ISA than PLA.

The width of anaesthesia in the region of attached gingiva and oral mucosa was significantly greater in the ISA in comparison to the PLA. This finding could be attributed to better spreading of anaesthetic solution with the ISA regarding the characteristics of tissue at the site of injection (42). Cortical perforations of the alveolar bone allow access of the anesthetic solution to more alveolar crest and, passing through buccal plate nutrition canal foramina, anaesthesia of the supraperiosteal soft tissue (9, 43). Compared to the results from the study of Brkovic et al. (31) in healthy individuals, the width of anaesthetic field for ISA was greater in our study in DM patients. This finding could be attributed to an increased permeability of the gingival epithelium in DM patients (44) and subsequent better spreading of anaesthetic solution. Another possible explanation could be articainès farmacokinetics—fast penetration through gingival tissue and alveolar bone (45).

It has already been shown that local anesthetics with vasoconstrictors are rapidly absorbed into the systemic circulation after both ISA and PLA (9). Consequently, transient cardiovascular effects may occur (31). However, the literature reports inconsistent findings regarding the effects of ISA and PLA on heart rate and blood pressure (16, 31). The results of our previous study in healthy individuals by Biocanin et al. (16) demonstrated no significant changes in blood pressure or heart rate when ISA and PLA were administered using CCLADS. Similarly, Nusstein et al. (46) reported no significant cardiovascular alterations after computer-controlled delivery of a larger volume (1.4 mL) of Art + Ep for PLA in healthy volunteers. In contrast, Smith and Pashley (47), in an experimental study on dogs, observed a significant increase in heart rate and a decrease in blood pressure after PLA administered with a high-pressure syringe.

In the present study involving patients with DM, cardiovascular parameters remained generally stable during and after administration of both ISA and PLA for extraction of the maxillary lateral incisor and mandibular first premolar. Although several statistically significant intergroup differences were observed for systolic blood pressure and mean arterial pressure during extraction of the maxillary lateral incisor, these differences were mostly related to slightly higher median values in the ISA group and were not accompanied by marked hemodynamic fluctuations. In addition, significant differences in some parameters were already present before anaesthesia administration, suggesting that baseline group characteristics may have contributed to these findings. During extraction of the mandibular first premolar, no statistically significant intergroup differences were observed for systolic blood pressure, diastolic blood pressure, mean arterial pressure, or heart rate.

Heart rate values showed only minor variations throughout the observation period in both anaesthesia groups. Although slightly higher median heart rates were observed in the PLA group during extraction of both teeth, small but statistically significant transient changes from baseline were observed during anaesthetic administration, however, these changes were not considered clinically relevant. Minor transient fluctuations in cardiovascular parameters observed during anesthetic administration may be associated with anxiety, stress related to needle insertion, and activation of the autonomic nervous system (48). The absence of pronounced cardiovascular responses in our study may also be related to the slow and controlled administration of anesthetic with epinephrine using CCLADS, resulting in slower systemic absorption of the vasoconstrictor. Taken together, these findings support the assumption that ISA and PLA administered with CCLADS do not induce clinically relevant cardiovascular disturbances in patients with controlled DM.

## 5 Limitations

Several limitations of the present study should be acknowledged. First, the study did not include a healthy control group, which would have enabled direct comparison of the anesthetic response and cardiovascular effects of ISA and PLA between patients with controlled type 2 DM and individuals without diabetes. However, the primary aim of this study was to evaluate and compare the efficacy and hemodynamic effects of two computer-controlled anaesthetic techniques specifically in patients with controlled type 2 DM, who represent a clinically relevant population requiring careful evaluation of local anaesthetic administration.

Second, the study was conducted at a single centre, which may limit the generalizability of the findings to other clinical settings and patient populations. In addition, only two tooth types were evaluated (maxillary lateral incisors and mandibular first premolars); therefore, the results may not be directly applicable to other teeth with different anatomical characteristics.

Finally, although cardiovascular parameters were monitored during and after anaesthetic administration, longer-term follow-up was not performed. Future multicentre studies including both diabetic and healthy control groups, as well as a broader range of dental procedures, are warranted to further confirm these findings.

## 6 Conclusion

The results of this study demonstrated that both ISA and PLA administered with CCLADS were safe and clinically effective anesthetic techniques in patients with controlled Type 2 DM, without clinically relevant cardiovascular alterations. ISA provided significantly longer duration and wider distribution of soft tissue anaesthesia compared with PLA, which may support its use as a reliable option for tooth extraction in diabetic patients.

## Data Availability

The datasets presented in this study can be found in online repositories. The names of the repository/repositories and accession number(s) can be found in the article/Supplementary Material.
